# Understanding the Experiences of Physical Activity, Body Image, and Quality of Life in Young Adult Males Living with and beyond Cancer

**DOI:** 10.3390/curroncol31080348

**Published:** 2024-08-15

**Authors:** Tana Dhruva, Jenna A. P. Sim, Chad W. Wagoner, Sarah J. Kenny, David M. Langelier, S. Nicole Culos-Reed

**Affiliations:** 1 Faculty of Kinesiology, University of Calgary, Calgary, AB T2N 1N4, Canada; 2 Ohlson Research Initiative Cumming School of Medicine, University of Calgary, Calgary, AB T2N 1N4, Canada; 3 Faculty of Arts, School of Creative and Performing Arts, University of Calgary, Calgary, AB T2N 1N4, Canada; 4 Department of Clinical Neurosciences, Division of Physical Medicine and Rehabilitation, Cumming School of Medicine, University of Calgary, Calgary, AB T2N 1N4, Canada; 5 Department of Medicine, Division of Oncology, Cumming School of Medicine, University of Calgary, Calgary, AB T2N 1N4, Canada

**Keywords:** exercise oncology, physical activity, body image, quality of life, YA males

## Abstract

For young adults (YAs), a cancer diagnosis and subsequent treatments may result in physical changes that can negatively impact body image (BI) and health-related quality of life (HRQL). Physical activity (PA) is an evidence-based tool found to impact both BI and HRQL. However, most research has focused on the perspectives of older adults with breast or prostate cancer. No research has explored the experiences of PA, BI, and HRQL in YA males affected by cancer. A qualitative study was designed for YA males diagnosed with cancer between the ages of 20 and 39 years. Eligible participants were recruited through pre-existing exercise oncology studies, support organizations, and social media. Semi-structured interviews were conducted to understand participants’ experiences of PA, BI, and HRQL. All interviews were transcribed verbatim and analyzed using interpretive description. The participants were YA males (*n* = 7) with a mean age of 32.7 ± 4.0 years. Themes included a loss of identity due to cancer, building autonomy and identity using PA, and the “should” behind BI. PA for YA males living with and beyond cancer may support them in rebuilding their identity and BI. The development of exercise oncology resources for YA males may consider addressing BI through education or exercise prescription programs, with the goal of enhancing HRQL.

## 1. Introduction

Approximately 2000 adolescent and young adults (AYAs) are diagnosed with cancer each year in Canada [1]. Of those, young adults (YAs) aged 20–39 years are most commonly diagnosed with thyroid cancer, breast cancer, Hodgkin lymphoma, non-Hodgkin lymphoma, testicular cancer, and melanoma [2]. Both the diagnosis and subsequent treatments often result in negative physical and psychosocial effects. Specifically, YAs may suffer from anxiety surrounding their cancer, fear of reoccurrence, depressive symptoms, and reduced self-esteem [3]. In addition, physical changes include impaired daily functioning, hair loss, weight gain/loss, reduced muscle mass, fatigue, and scarring [3]. The physical changes in particular may contribute to negative perceptions of one’s body image (BI). 

YAs affected by cancer face unique challenges in their experiences of BI during important developmental milestones [4]. Specifically, YAs’ cancer experiences disrupt milestones such as gaining personal independence, building relationships, developing their educational and career goals, and undergoing physical and functional changes, all of which may contribute negatively to their BI [4,5,6]. The resultant BI dissatisfaction in turn may lead to a decrease in HRQL [3]. Specifically, cancer-related BI dissatisfaction is associated with greater levels of psychological distress including depression, anxiety, and post-traumatic stress [7]. 

While the research on BI perceptions in YAs is growing, the focus has primarily been on the perspective of younger women with breast cancer [8,9,10,11,12]. Thus, there is a need to better understand the male perspective of BI within the YA cancer population. The limited literature on male BI has found potential differences of BI perceptions between males and females [13,14]. Specifically, females often place an emphasis on the physical perception of their bodies, whereas males may focus more on their functionality and physical capabilities with regards to muscular strength changes [13,15]. Females are also more likely to experience negative BI surrounding their physical appearance and weight in comparison to males [4,14]. Conversely, males may face unique psychological distress as a result of their negative BI tied to their perception of muscle dysmorphia and the impact of their scarring/disfigurement [7]. These negative BI perceptions in YA males living with and beyond cancer present not only a need to understand the impact of BI on HRQL, but also to improve care and provide resources for YA males with negative BI. One such resource that has been studied is PA. 

PA is an evidence-based intervention for improving HRQL in individuals living with and beyond cancer [16,17,18,19,20,21]. PA has been associated with improving HRQL by reducing cancer-related symptoms (i.e., fatigue, lymphedema, etc.), improving psychosocial well-being (i.e., anxiety, depressive symptoms, etc.), and improving physical functioning across all cancer types and populations [22,23]. PA is also associated with more positive BI, encouraging individuals to focus on functionality rather than perceptions of their bodies [21]. Despite this, less than 10% of individuals are meeting physical activity guidelines while receiving treatment, and individuals transitioning into survivorship spend 66% of their time sedentary [18,24]. To date, most of the literature on PA, BI, and HRQL has focused on adult females with breast cancer [25,26,27]. Research on males is limited and primarily in older males with prostate cancer [15,28,29]. Given the limited evidence, specifically in YA males, the purpose of this study was to explore the experiences of PA, BI, and HRQL for YA males affected by cancer.

## 2. Materials and Methods

### 2.1. Participants and Recruitment

Eligibility for this study included English-speaking YA males (cis or trans) diagnosed with cancer between the ages of 20 and 39 years, across Canada. There was no restriction on tumor/cancer type, stage, or whether they were in the pre-treatment, active treatment, or survivorship phase. Participants were primarily recruited from pre-existing exercise oncology studies at the Health and Wellness Lab at the University of Calgary, including Alberta Cancer Exercise (ACE; NCT02984163), Exercise for Cancer to Enhance Living (EXCEL; NCT04478851), and Yoga for Young Adults Affected by Cancer (YYA; NCT04478851). Furthermore, we reached out to community, clinical, and academic contacts to disseminate study information and utilized social media platforms to recruit potential participants. Participants were recruited from July to December 2023.

### 2.2. Data Collection and Analysis 

Semi-structured interviews were designed to understand participants’ perceptions and experiences of their PA, BI, and HRQL in relation to their cancer diagnosis and treatment. Interviews were conducted online through ZOOM^TM^ (Version 6.0.11) or over the phone. As per the interpretive description methodology, guided with a constructivist epistemology, the analysis included verbatim transcription of interviews, the generation of codes, and the development of themes from patterns in the interviews [30]. Rigor was maintained through the assistance of a critical friend (J.A.P.S.), the use of a reflexive journal, and an audit trail.

## 3. Results

### 3.1. Demographics

Seven participants consented to and participated in a semi-structured interview. Participant demographics and clinical characteristics are summarized in Table 1 and Table 2. The participants’ age at diagnosis ranged from 28 to 38 years, with a mean age of 32.7 ± 4.0 years. 

### 3.2. Interview Results 

The resultant themes from the interviews were as follows: (1) a loss of identity due to cancer, (2) building autonomy and identity using PA, and (3) the “should” behind BI. The themes and representative quotes are described below. Additional quotes are reported in Table A1 in Appendix A. 

#### 3.2.1. Theme 1: Loss of Identity Due to Cancer

Participants experienced changes to their physical abilities following their cancer experience. Most commonly, participants reported symptoms such as fatigue and neuropathy, which affected their body’s ability to function as it used to. These physical changes interfered with their activities of daily living and were associated with diminished physical well-being.

*Not as- able to be as active as I was before the cancer and then some of the side effects from the some of the chemotherapy that caused neuropathy for me in my hands and feet. So that makes the balance in particular for things like paddle boarding more challenging.*

P07

Due to their declining physical ability and performance, YAs noticed differences in their ability to engage in their usual PA. For many, participating in PA defined their identity.

*‘cause my running performance was declining kind of rapidly. And I was like, what is this? This doesn’t make sense. Is this getting old? Can I just not run a 25-min 5K anymore?*

P02

Along with being unable to participate in their usual levels of PA, participants showed an awareness of what their bodies were or were not able to achieve. In the process of adapting PA to their changing physical abilities, participants expressed a loss of their identity prior to cancer. The loss of the “old me” created a sense of frustration and dissatisfaction.

*It’s sort of frustration, sense of loss of not being the old me and frustration that I’m not, yeah, where I’d want to be or hoped I could be.*

P07

Finally, these changes in physical function and their changing BI and identity were associated with their mental well-being.

*Somewhere in there, I have like some other surgical issues that are, you know, like causing me to kind of, like be mentally drained and frustrated.*

P01

Overall, the cancer experience changed YA males’ PA levels, with these limitations (i.e., reductions in PA and exercise engagement) further contributing to their loss of identity associated with performing these activities prior to being diagnosed. 

#### 3.2.2. Theme 2: Building Autonomy and Identity Using PA

For some participants, an initial motivator for engaging in PA was a desire to improve their physical functioning and overall HRQL. Specifically, the inability to carry out the tasks that they used to and the loss of body function that they experienced was a trigger for participants to focus on their health.

*So, pre- I didn’t really care much about my health. I ate whatever, didn’t really exercise, and then the fact that I need to do some exercise post-surgery to recover, that was an initial trigger toward continuing with exercise to this day, as irregular as it is. But it is more than I was doing five years ago.*

P05

For some participants, weight management was another motivator to engage in PA as they believed it would improve their health. YA males believed that by finding a “balance” with their weight, they would become healthier.

*And right now, it was it’s about getting healthy again. And so prior, like right at the beginning of treatment, they’re like you need to get up to weight and now I’ve gotten to weight, so now I don’t want to get overweight.*

P03

Furthermore, by reaching a “healthy” weight, participants felt as though they would be able to align further with their identities prior to cancer. For one participant, this is exemplified in how he believes that by reducing his weight, he will live longer and be able to experience more with his daughter. This was important for him as it was pivotal to his identity as a parent.

*Staying alive is kind of a big motivating factor in losing weight and being healthy and being around to, you know, have my daughter when she’s 16 to tell me to f-off. Like, that’s a life experience that I’m going to want to have, right?*

P01

PA was a means of establishing autonomy in their lives when it was otherwise lost because of cancer. Setting goals to be able to measure their progress fostered their sense of control, providing a sense of achievement that further reinforced their autonomy.

*With the body weight [exercise routine] I can definitely see, oh, I could only do like 4 push ups last month, now I’m up to five. OK, now let’s move on to like the next difficulty level. And that kind of feels satisfying that I’m meeting some sort of goal and making progress in my physical fitness.*

P05

PA allowed the participants to feel empowered and appreciative for what their bodies were able to do rather than focusing on the limitations and the decline in their abilities. This was reported to enhance both physical and mental well-being.

*I think the exercise is really, yeah, it’d be a bit like, just awe and appreciation of like, what my body is capable of. Because even like 10 years ago I’ve been like, I’m never going to run. I’m never going to be able to run more than like around the block. It’s like, no, actually you can run really fing far if you want to.*

P02

Participants used PA to rebuild a sense of control by focusing on what they could do physically. By engaging in PA, participants felt a sense of accomplishment and pride for what their bodies were capable of, facilitating their new “view of self”. 

#### 3.2.3. Theme 3: The “Should” behind BI: Perceiving One’s Body Based on What They Should Look Like

Participants reported changes to their physical appearance such as having a colostomy bag, gaining/losing weight, losing hair, or not being able to fit into clothes they once could. The changes that they experienced were reflected in negative feelings toward their body.

*Yeah, so hair loss was a big one for me. I still have a little bit, but used to have a lot more. So that was, I mean it kind of slowly, like seeing it at the beginning, first to go that was that was a bit of a struggle for me because I didn’t want to lose the hair.*

P03

The impact of negative BI on the mental well-being of YA males living with and beyond cancer was evident, with one participant indicating that it was a topic that they “would talk to a therapist about” (P01). Some participants also mentioned that the perception of their body impacted their day-to-day functioning.

*Well, it’s, I don’t know, maybe made me a bit more depressed, that I‘ve gained this weight.*

P04

The negative feelings participants experienced were often due to being unable to meet an expectation of what their body “should” look like and what they think is a socially desirable body type. The idea of being socially desirable was often related to their ideas of masculinity.

*Because I want it to be like all of the boy band stomachs from the 90s, man. I wanna look like that. Unfortunately, it’s a toxic trait, I guess.*

P02

How YA males viewed their body was also dependent on how they thought they were perceived by others. Feeling self-conscious about their body occurred when they thought, in particular, that they were being perceived negatively by others.

*Worrying about what other people think or how what other people’s perceptions are wearing out when you don’t the shirts too tight or don’t look bit bigger or bit overweight.*

P07

Taken together, these negative feelings surrounding their physical appearance, alongside the perceived judgment by others, often served as a motivator to change how their bodies looked. 

## 4. Discussion

Overall, our findings are the first steps in exploring the experiences of YA males living with and beyond cancer in relation to PA, BI, and HRQL. YA males experienced treatment-related symptoms and side effects that impaired their ability to engage in PA. YA males reported being unable to participate in regular PA or other activities of daily living that defined their sense of self. This was reported as a loss of the “old me”, or their pre-cancer identities. This perceived loss of self is supported by qualitative assessments in AYAs impacted by cancer, which also document a similar shift in their identity and self-concept [31,32]. In the current work, many of the YA males were active prior to their cancer diagnoses, participating in structured PA or through work/sports. Being unable to engage in these activities, compared to their previous active selves and in a similar capacity to what they were used to, led to a heightened sense of disappointment and frustration. These findings are similar to an earlier work examining AYAs’ transition back into PA following their treatment, where changes in PA levels were associated with discouragement and demotivation [33]. 

For many YA males, the decline in their physical functioning coupled with the changes to their PA due to treatment became a motivator for re-integrating PA into their lives. Engaging in PA provided them with a sense of structure and routine, consistent with previous research illustrating that many AYAs use PA to feel “normal” again and find control in the uncontrollable after cancer [33]. For participants, PA was a tool to exercise their autonomy, and similar to previous studies with adult men with prostate cancer, PA allowed them to exercise control and feel empowered [34]. Participation in PA also caused participants to report a shift in focus from their physical appearance to the overall functioning of their body. Congruent with these findings, it has been found that physically active individuals have a greater physical self-concept in comparison to those who are less active [20]. In the present study, participants experienced an appreciation for their bodies after re-engaging in PA following their cancer. Furthermore, the feeling of empowerment upon engaging in PA was in relation to the experience of autonomy as well as the achievement of goals. Studies have found that the concept of goal setting and subsequent achievement by participants moderated the relationship between PA and the physical self-concept [20]. 

While PA was a tool to build autonomy, it was also a tool to manipulate their physical appearance. This is demonstrated in the literature reporting on AYAs’ desire to use PA to “offset” the changes they experienced due to cancer [33]. This desire for physical change aligns with our findings, wherein YA males used PA to alter aspects of their appearance impacted by the cancer. Instances of this included weight management and building muscle. The need to change their physical appearance may be linked to the unhappiness and dissatisfaction YA males experienced following the physical changes they underwent from cancer treatment. Similarly, previous research in AYA populations found that approximately 59.6% of people between the ages of 21 and 29 and 61.5% of those between 30 and 39 years expressed negative feelings about how they look [35]. For YA males, the dissatisfaction with their physical appearance may be related to the departure from masculine norms within this specific age group upon being diagnosed with cancer. While our findings did not explicitly reveal this, previous research highlights the decline in a male’s sense of masculinity due to the effects of prostate cancer treatment [36]. Future work investigating YA males’ perspectives of masculinity in relation to BI is warranted.

## 5. Conclusions

Despite comprehensive recruitment efforts, the sample for the current study was small and drawn primarily from existing PA studies. Struggles recruiting YAs for research have been highlighted in oncology settings [37]. Future work must continue to employ targeted recruitment strategies and work with YA male participants to determine the best means to encourage participation. Using a participant-oriented research approach and building clinical team support to increase awareness of study opportunities for YA males may improve recruitment in future work.

While we focused on YA males, the relatively older age range (above 30 years) for the current sample reduces the generalizability for those on the younger end of the spectrum. BI can vary across the age range for YAs [32], and thus, future work must continue to examine these relationships in younger males (20–30 years). Lastly, while this study sought to understand the perspectives of all YA males living with and beyond cancer, the majority of participants self-identified as “White”, with only one participant identifying as “East Asian”. As such, it should be recognized that the perspectives from a racially diverse sample may differ from what is presented in this study. 

Despite these limitations, this study adds to our preliminary understanding of PA, BI, and HRQL in YA males living with and beyond cancer. The findings suggest the importance of PA in rebuilding identity, BI, and HRQL in YA males affected by cancer. Future work can build upon these findings to consider how PA resources (education and programs) must be tailored to address BI and HRQL for YA males living with and beyond cancer. For example, resources that address the BI concerns in YA males may support their willingness to participate in PA. Studies have shown that tailored informational materials for YAs increased PA levels among inactive YAs, demonstrating their potential as a cost-effective intervention to enhance PA levels [38]. In addition, tailored exercise programming addressing both BI and masculinity concerns, while also cultivating a sense of autonomy, may be considerations within future programming. This may include providing participants with a choice of activity such as participation in sports or performing activities which may address physical changes following cancer (e.g., resistance training) whilst improving physical functioning.

Therefore, building upon these results, continued investigation may help to inform the creation of appropriate PA and exercise resources for the YA male population living with and beyond cancer to ultimately support positive BI and HRQL outcomes.

## Figures and Tables

**Table 1:** Participant demographics and clinical characteristics (*n* = 7).

Variable	Number (%)
Age at Diagnosis: Mean ± SD, years *	32.7 (4.0) [range: 28.0–38.0]
Sexual Identity/Orientation ^†^	
Gay	1 (14.3)
Questioning	1 (14.3)
Straight (Heterosexual)	5 (71.4)
Race ^†^	
East Asian	1 (14.3)
White	6 (85.7)
Residence ^†^	
Alberta	6 (85.7)
Northwest Territories	1 (14.3)
Rural/Urban ^†^	
No Information Provided	1 (14.3)
Urban Area	6 (85.7)
Cancer Type ^†^	
Colon	1 (14.3)
Colon and Brain	1 (14.3)
Gastrointestinal Stromal Tumor (GIST)	1 (14.3)
Hairy Cell Leukemia	1 (14.3)
Follicular Lymphoma	1 (14.3)
Hodgkin’s Lymphoma	1 (14.3)
Testicular	1 (14.3)
Treatment Status ^†^	
Currently Receiving Active Treatment	2 (28.6)
Completed Treatment	5 (71.4)
Employment ^†^	
Disability	3 (42.9)
Full-Time	3 (42.9)
Temporarily Unemployed	1 (14.3)
Highest Education Level Achieved ^†^	
Completed Graduate School	1 (14.3)
Completed University/College	4 (57.1)
Some Graduate School	1 (14.3)
Some University/College	1 (14.3)
Marital Status ^†^	
Common-Law	2 (28.6)
Married	3 (42.9)
Single	2 (28.6)

Notes: * mean (standard deviation) [range]; ^†^ *n* (%).

**Table 2:** Participant characteristics (*n* = 7).

Participant #	Age at Diagnosis	Current Age	Cancer Type	Treatment Status	Biological Sex	Pronouns	Sexual Identity/ Orientation
P1	37	40	Gastrointestinal Stromal Tumor (GIST)	Completed	Male	He/Him/His/Himself	Straight (heterosexual)
P2	35	39	Hairy Cell Leukemia	Completed	Male	He/Him/His/Himself	Questioning
P3	28	28	Hodgkin’s Lymphoma	Chemotherapy	Male	He/Him/His/Himself	Straight (heterosexual)
P4	38	38	Follicular Lymphoma	Immunotherapy	Male	He/Him/His/Himself	Gay
P5	32	37	Colon	Completed	Male	He/Him/His/Himself	Straight (heterosexual)
P6	30	38	Testicular	Completed	Male	He/Him/His/Himself	Straight (heterosexual)
P7	29	36	Colon and Brain	Completed	Male	He/Him/His/Himself	Straight (heterosexual)

## Data Availability

The original contributions presented in this study are included in the article; further inquiries can be directed to the corresponding author.

## Appendix A

**Table A1:** Additional participant quotes.

Theme:	Example Quotes
Loss of identity due to cancer.	*I’m just like physically tired, like all the time. It’s just kicking myself to get myself moving.* P01 *I will pass hanging out with friends just because I know it’s not worth pushing the body.* P03 *I felt and what I could physically do, versus now, I have more limitations.* P07 *I wasn’t able to participate at be as active, played team sports and went to the gym, worked out more before my diagnosis and then treatment and haven’t been able to get back to that level.* P07 *Yeah, I think it’s impacted my mental health in a negative way. There’s so many things that you can’t control from cancer and we can’t fully control our body as fit and whiz, but sort of losing the ability to be as active or to stay in is the best ship is the shape that I’d want to be in has been a challenge mentally.* P07 *It’s feeling like I can’t or I don’t have the energy or the inability to do it more so than not liking how it look while I do those things.* P07
Building autonomy and identity using physical activity.	*Well that physical activity should lead to a healthier lifestyle as far as increasing my endurance, hopefully causing less pain in your knees. And, you know, just overall well-being like that physical activity will keep you, you know, to have less likelihood of maybe hurting my back when I when I pick her [participant’s daughter] up, you know, or you know, just things like that from injury, I guess.* P03 *I 100% agree uhm that like for the longevity my life, I need to lose weight. Like it’s a weird thing. Like, I know everybody says it, but like once you have kids, like your whole like, priority list changes as far as like what it is that you want and all that stuff. It no longer is you. You’re thinking of someone else. And I would hate like, you know all the studies as you know how it goes like a kid who grows up without a parent in the house, like they could end up on crack [laughs].* P03 *The worry’s not so much I guess in dying that’s more of a heart disease from a history on the history is probably the driving factor on that you know degradation of physical performance let’s say is probably the more pertinent worry from the cancer diagnosis and follow out.* P06
The “should” behind body image: perceiving one’s body based on what they should look like.	*Like, people always underestimate me due to like, you know, I’m quiet, I’m overweight, but like in majority of sports, I’ve always done fairly well. Like as far as like I’m not a professional or anything, but I’m fairly competent.* P01 *So, I was in shorts and T-shirt for a very long period of time with the uhm the chicken legs and all of that going on. So, I was none too pleased with that. So that was definitely, just definitely a body image thing of me wanting to put on the weight and the muscle because yeah, I was just getting horrendously skinny. And but then yeah, that that would have been the body image thing.* P03 *Like, if I can even, like, go to, like, out with friends or like, I don’t really go to the bars anymore because I’m like, self-conscious about my body pretty much.* P04 *So, it’s more aspiring to have the weight I had say, during grad school as opposed to 10 years later.* P05 *What I actually want to do is once in a while when I meet, I know my father-in-law or my cousin to be able to show off. ‘Hey look, I actually have muscles now’.* P05
